# Postproduction Approach to Enhance the External Quantum Efficiency for Red Light-Emitting Diodes Based on Silicon Nanocrystals

**DOI:** 10.3390/nano12234314

**Published:** 2022-12-05

**Authors:** Hiroyuki Yamada, Junpei Watanabe, Kazuhiro Nemoto, Hong-Tao Sun, Naoto Shirahata

**Affiliations:** 1 International Center for Materials Nanoarchitectonics (MANA), National Institute for Materials Science (NIMS), 1-1 Namiki, Tsukuba 305-0044, Japan; 2 Graduate School of Chemical Sciences and Engineering, Hokkaido University, Sapporo 060-0814, Japan; 3 Department of Physics, Chuo University, 1-13-27 Kasuga, Bunkyo, Tokyo 112-8551, Japan

**Keywords:** silicon nanocrystals, light-emitting diode, external quantum efficiency

## Abstract

Despite bulk crystals of silicon (Si) being indirect bandgap semiconductors, their quantum dots (QDs) exhibit the superior photoluminescence (PL) properties including high quantum yield (PLQY > 50%) and spectral tunability in a broad wavelength range. Nevertheless, their low optical absorbance character inhibits the bright emission from the SiQDs for phosphor-type light emitting diodes (LEDs). In contrast, a strong electroluminescence is potentially given by serving SiQDs as an emissive layer of current-driven LEDs with (Si-QLEDs) because the charged carriers are supplied from electrodes unlike absorption of light. Herein, we report that the external quantum efficiency (EQE) of Si-QLED was enhanced up to 12.2% by postproduction effect which induced by continuously applied voltage at 5 V for 9 h. The active layer consisted of SiQDs with a diameter of 2.0 nm. Observation of the cross-section of the multilayer QLEDs device revealed that the interparticle distance between adjacent SiQDs in the emissive layer is reduced to 0.95 nm from 1.54 nm by “post-electric-annealing”. The shortened distance was effective in promoting charge injection into the emission layer, leading improvement of the EQE.

## 1. Introduction

Solution-processed quantum dot light-emitting diodes (QLEDs) using cadmium- and lead (Pb)-free quantum dots (QDs) as an emissive layer have gained much attention due to the potential applications such as wearable, lightweight, and environmental-friendly optoelectronic devices that could be produced at low-cost. At present, QLEDs that meet the benchmark of 20% external quantum efficiency (EQE), which is comparable to the energy conversion efficiency of a mercury lamp, are limited to models that use indium phosphide QDs as an optically active layer [1]. In case of red-QLEDs, the possible QD alternatives are copper indium sulfide (CuInS_2_) [2], ZnS-AgInS_2_ (ZAIS) [3], Pb-free perovskite nanocrystals [4], and silicon (Si) [5,6,7,8,9,10,11,12,13,14,15,16,17,18,19,20,21], but the EQE values reported for these QLEDs are still low, such as 7.8% for CuInS_2_ [2], 2.2% for ZAIS [3], 0.3% for Pb-free perovskite [4], and 6.2% for Si [17].

Si is the second most abundant element on earth and has no environmental toxicity [22]. The effect of the quantum confinement (QC) manifests in crystalline Si particle smaller than 5 nm in diameter [23]. Varying the SiQD diameter between 1.1 nm and 11.8 nm allows the photoluminescence (PL) peak to be tuned over a wide spectral range between 535 nm and 1050 nm [24,25,26]. Termination of surface atoms of the QDs with hydrocarbon chains via covalent Si-C linkage is known to give a dramatic increase in the radiative recombination rate to enhance PLQY up to 60% [27,28,29,30]. Possible mechanism includes inactivation of nonradiative channels [31] and transformation of Si nanocrystal into the fundamental direct bandgap semiconductor via a concerted action of QC and tensile strain [32,33]. Then SiQDs are not suitable as emission layers for phosphor-type LEDs due to their low absorbance in the visible wavelength region. In contrast, since charge carriers are injected from the electrode, the EQE is theoretically dominated by PLQY, which may be compatible with current-driven device architecture. As expected, the first Si-QLEDs showed a near-infrared (NIR) electroluminescence (EL) character of 8.6% EQE and its spectrum was centered at 853 nm [8]. Since then, much effort has been devoted developing the visible-light emitting Si-QLEDs, such as 6.2% for red EL [17], 0.12% for yellow-orange EL (i.e., 590−630 nm) [19], and 0.03% for white EL spectra [34].

In general, colloidal stability of QDs is given by organic ligands with long molecular chains, such as octadecane, oleic acid, and oleylamine, whereas short ligands are suitable for increasing carrier mobility in the optically active layer of QLEDs [35]. Therefore, ligand-exchange, in which the long molecular chains are replaced by short counterpart, is a common strategy in the QD-based device fabrication process. For SiQDs, the organic ligands (i.e., decane and octadecane) are covalently bonded to outermost Si atoms, making ligand exchange impossible. Alternative methods are limited to the use of ligands with short molecular chains in the hydrosilylation reaction step. Liu et al. used to cover the SiQD with octyl monolayers for shortened interparticle distance in the emission layer, and succeeded in achieving an EQE of 6.2%, the highest ever achieved for red-emitting Si-QLEDs [17]. Since linear hydrocarbons used as ligands have low electrical conductivity, the use of short ligands would increase the charge injection efficiency into the optically active layer and improve the EQE compared to long ligands. In other words, the shorter the interparticle distance, the more efficient charge injection can be expected and the higher the EQE. Here, we report a straightforward fabrication method called post-electric-annealing, in which a voltage bias is continuously applied to the Si-QLED to shorten the interparticle distance between QDs in the emitting layer, thereby increasing the EQE.

## 2. Materials and Methods

### 2.1. Reagents and Materials

Triethoxysilane (TES) was purchased from Tokyo Chemical Industry Co., LTD. (Tokyo, Japan). Colloidal ink of zinc oxide (ZnO), 1-decene, hydrochloric acid (HCl) and molybdenum (IV) oxide (MoO_3_, 99.97% trace metal basis) were purchased from Sigma-Aldrich and used as received. 4,4′-Bis(carbazole-9-yl)biphenyl (CBP, 99.9% trace metals basis) was purchased from Luminescence Technology Corp. Electronic-grade hydrofluoric acid (HF, 48% aqueous solution) was purchased from Kanto Chemical Co., INC. Toluene (HPLC-grade), chloroform, ethanol (99.5), methanol, and Zn powder were purchased from Wako Chemical. Milli-Q water (resistivity = 18.2 MΩ·cm) was obtained by Sartorius water purification system (arium 611 UV).

### 2.2. Preparation of SiQD Ink

SiQDs were synthesized via a two-step process reported in our previous papers [36]. TES was hydrolyzed by an aqueous HCl solution at pH 3 under Ar gas flow. The filtered solution produced a white precipitate, which was washed until its pH reached 7. After drying the white precipitates overnight under vacuum, they, i.e., (HSiO_1.5_)_n_, were transferred to a furnace and heated to 1050 °C in a 95%/5%-Ar/H_2_ atmosphere and held for 2 h for thermal disproportionation to obtain a dark-brown powder of SiO_x_-encapsulated SiQDs. After cooling down to room temperature, 300 mg of the dark-brown powder was mechanically ground using an agate mortar equipped with a pestle. The resulting fine powder was stirred in an acidic solution containing 8 mL ethanol and 16 mL 48%-HF solution for 90 min to liberate QDs from the SiO_x_ matrix and simultaneously terminate the SiQD surface with hydrogen atoms (H-SiQD). The H-SiQDs were collected by centrifugation at 10 °C and 15,000 rpm. The H-SiQDs were then subjected to thermal hydrosilylation of 1-decene at 175 °C to obtain decane-terminated SiQDs (De-SiQDs). After removing an unreacted 1-decene by heating the reaction solution in vacuum, they were redispersed in chloroform. Next, the chloroform solution of De-SiQDs was subjected to the HPLC process. The purified SiQDs were redispersed in toluene at a concentration of 10 mg/mL and used as a QD ink.

### 2.3. Device Fabrication

Si-QLEDs were fabricated on a soda-lime glass substrate. A thin film of ITO uniformly sputtered on the glass with 130 nm thickness gave a resistivity of 10–14 Ω/sq. The chemical etching was performed using HCl and Zn powder to process the ITO thin film into narrow strips of 2 × 20 mm. Then, the sample was ultrasonicated with Milli-Q water, acetone, ethanol, and isopropanol, in that order. After drying, the ITO surface was irradiated under N_2_ flow at a pressure of 10^3^ Pa for 30 min using a VUV lamp (Ushio Inc., Tokyo, Japan) to remove hydrocarbons by oxidation. The sample was immediately transferred into the glovebox filled with Ar gas. In the first step of multilayer formation, the colloidal ink of ZnO was spin-coated at 2000 rpm for 60 s. After drying at 120 °C, the QD ink was spin-coated at 1000 rpm for 60 s. After removal from the glovebox, the sample was transferred to the vacuum evaporation chamber and the pressure was reduced until the vacuum reached 2 × 10^−5^ Pa. A 48-nm-thick CBP layer and 41-nm-thick MoO_3_ were thermally deposited on the QD-covered surface in that order. Finally, a 227-nm-thick Al thin film as a top electrode was deposited.

### 2.4. Characterization

An X-ray diffraction (XRD) experiment was carried out on MiniFlex600 diffractometer (Rigaku, Tokyo, Japan) with Cu Kα X-ray source (λ = 1.5418 A, 20 kV and 30 mA). The XRD pattern was collected in the range 20° ≤ 2θ ≤ 65° with a step size of 0.02° per step and scan speed of 0.1° per minute, by one-dimensional X-ray detector D’teX Ultra 250. The nanoparticle with dispersion medium (SiQDs in toluene, 10 μL) was dropped and dried onto a non-diffractive Si sample holder. The crystallite size was evaluated from full width at half maximum of diffraction peaks using Scherrer’s equation. The synthesized QD was characterized by high-resolution transmission electron microscopy (HR-TEM, JEOL JEM-2100F, Tokyo, Japan) operated at 200 kV. Optical absorbance spectrum of the De-SiQD dispersed in toluene was measured by UV-vis spectrophotometer (JASCO V-650, Tokyo, Japan). PL spectrum was measured by a modular double grating Czerny–Turner monochromator and iHR 320 emission monochromator (1200 lines/mm of gratings) coupled to a photomultiplier tube (PMT) on a NanoLog Horiba Jovin Yvon spectrofluorometer with a 450 W xenon arc lamp. The spectral resolution of the system was around 0.3 nm. A cut filter of 495 nm-light was placed in front of the monochromator-PMT setup. The absolute PLQY was measured using QY measurement system C9920-02 from Hamamatsu Photonics Co., Ltd. with a 150 W xenon lamp coupled to a monochromator for wavelength discrimination, an integrating sphere as a sample chamber, and a multichannel analyzer for signal detection. SiQDs dispersed in toluene were used for PL measurements. Ultraviolet photoelectron spectroscopic (UPS) spectra were measured by SigmaProbe (Thermo Fisher Scientific, Waltham, MA, USA). A photoelectron yield spectrum (PYS) was measured by a model AC-3 (RIKEN KEIKI Co., Ltd., Tokyo, Japan). The samples for both UPS and PYS measurements were thin-film forms of ZnO and De-SiQD spin-coated on ITO-coated glass substrate for antistatic electricity which were made in a manner similar to the device fabrication process. For device characterization, a calibrated Si photodetector (S1336-8BQ, Hamamatsu Photonics K.K., Shizuoka, Japan) coupled with a Keithley 2400 was used.

### 2.5. Observation and Analysis

Cross-sectional scanning electron microscopic (SEM) images of the multilayer structure were obtained by ZEISS Auriga Laser (Carl Zeiss, Oberkochen, Germany) combined with a focused ion beam (FIB) with a Ga^+^ ion source. The FIB processing was performed for the carbon protection coating (deposited from carbon gas) and subsequent making of crevices to reveal the cross-sectional observation surface. Next, the microstructure was observed by SEM operated at 1 kV under a 54° gradient condition. Cross-sectional high-angle annular dark field scanning TEM (HAADF-STEM) images were obtained by Tecnai Osiris (FEI, Hillsboro, OR, USA). Before FIB processing with a Ga^+^ ion source (FB-2100, Hitachi, Tokyo, Japan), the substrates were coated with a chromium (Cr)-containing oil-based ink to protect the surface. Next, FIB processing was performed for the tungsten (W) protection coating (deposited from W(CO)_6_ gas) and subsequent preparation of the cross-sectional samples. Each sample was then mounted on a copper (Cu) FIB lift-out grid and thinned to approximately 100 nm. The prepared cross-sectional ultrathin samples were analyzed using HAADF-STEM operated at 200 kV.

### 2.6. Calculation of EQE and Optical Power Density

EQE and optical power density were calculated assuming that EL has a Lambertian emission profile. EQE is expressed as the ratio of the number of radiated photons to the number of injected electrons per unit time $I_{d}\left(V\right)/\left|e\right|$, where $I_{d}\left(V\right)$ is current through device when applied voltage V and e is the electron charge. EQE is represented by the following equation [37]:

(1) $$\mathrm{EQE}\;\left(\%\right)=\frac{N_{p}\left(V\right)\times \left|e\right|\times g}{I_{d}\left(V\right)}\times 100$$

where $N_{p}\left(V\right)$ is the number of photons collected by photodiode. Geometry factor g expresses when EL profile assumed to be Lambertian the ratio of the luminous flux emitted from LED to the luminous flux measured by photodiode, given by the following equation:

(2) $$g=\frac{a^{2}+L^{2}}{a^{2}}$$

where a indicates the radius of aperture of photodiode and L is distance between the light emitting surface of LED and photodiode. Here, the geometry factor was calculated as 2.49. $N_{p}\left(V\right)$ based on the actual observed EL spectrum EL(λ) and photodiode current $I_{p}^{m}$ is given by the following equation:

(3) $$N_{p}\left(V\right)=\int_{\lambda_{i}}^{\lambda_{f}}EL\left(\lambda \right)\times \frac{I_{p}^{m}}{I_{p}^{\prime }\left(\lambda \right)}d\lambda$$

where $I_{p}^{\prime }\left(\lambda \right)$ is expressed using Planck constant h, the speed of light in vacuum c, EL emission wavelength λ, and photodiode responsivity R(λ):

(4) $$I_{p}^{\prime }\left(\lambda \right)=\int_{\lambda_{i}}^{\lambda_{f}}EL\left(\lambda \right)\times \frac{hc}{\lambda }\times R\left(\lambda \right)d\lambda$$

Calculating $N_{p}\left(V\right)$ for all applied voltages and substituting it into Equation (1) gives voltage or current density dependent EQE characteristics. Optical power density was calculated as follows. The emitted power P(V) is calculated by following equation:

(5) $$P\left(V\right)=\int_{\lambda_{i}}^{\lambda_{f}}EL\left(\lambda \right)\times \frac{I_{p}^{m}}{I_{p}^{\prime }\left(\lambda \right)}\times \frac{hc}{\lambda }d\lambda$$

Then optical power density is expressed using P(V)

(6) $$\mathrm{Optical}\;\mathrm{power}\;\mathrm{density}\;\left(W/{\mathrm{cm}}^{2}\right)=\frac{P\left(V\right)}{A}$$

where A is the area of the light emitting surface.

## 3. Results

### 3.1. Hydrophobic SiQD Ink

**Figure 1:**
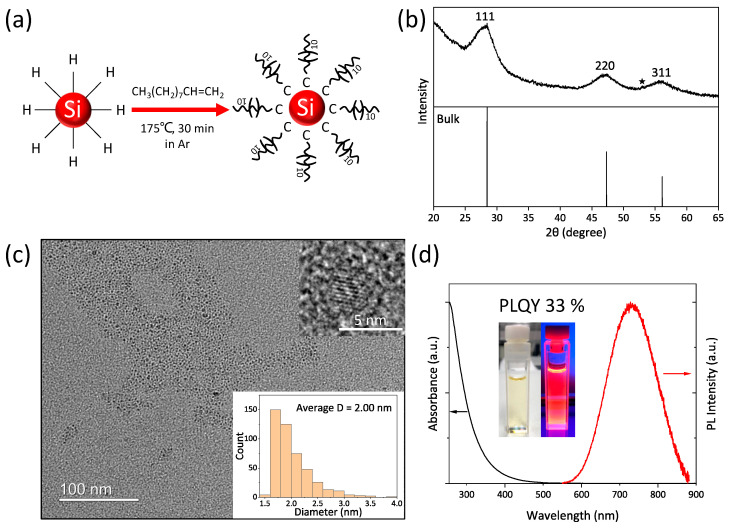
(**a**) Schematic illustration for preparation of the decane-terminated silicon quantum dot (De-SiQDs). (**b**) X-ray diffraction (XRD) pattern of De-SiQDs. The slight peak in near 53 degrees (shown by asterisk) is probably due to noise. (**c**) High-resolution transmission electron microscopy (HR-TEM) image with an inset of enlarged QDs and histogram of QD’s size distribution and (**d**) optical absorption and photoluminescence (PL) spectra of De-SiQDs dispersed in toluene. The inset is photographs of the toluene solution of De-SiQDs under the illumination of room light (left) and a 365-nm UV handy lamp (right).

Various synthetic routes have been reported to synthesize SiQDs with diamond cubic lattice structure [22,27,38]. In this work, we utilized the thermal disproportionation reaction of hydrogen silsesquioxane (HSQ), which was established by Veinot et al., as a synthetic method for SiQDs with high crystallinity and relatively narrow size distribution [38]. In many cases, commercially available HSQ is used to synthesize SiQDs, but HSQ can also be prepared by hydrolysis and condensation reactions of TES or trichlorosilane molecules [39,40,41]. H-SiQDs liberated from the thermally disproportionated HSQ (i.e., white powder of SiQD/SiO_x_ composite) were subjected to the thermal hydrosilylation of 1-decene, yielding De-SiQDs as shown in Figure 1a. Only De-SiQDs with PLQY greater than 30% were fractionated by HPLC separation process as in the conventional method [20]. Figure 1b shows a typical XRD pattern of the product after thermal hydrosilylation of 1-decene. The diffraction peaks at 2θ = 28, 47, 56° were indexed, respectively, to the (111), (220), and (311) planes of a diamond cubic Si lattice structure. As shown in Figure 1c, all observed dots were QDs with a round shape. The inset shows a histogram of the size distribution obtained by measuring the diameters of 462 dots. The average diameter of the QDs was found to be 2.0 nm, in good agreement with the Scherrer broadening analysis (~2.1 nm). The UV-vis absorption and PL spectra of De-SiQDs dispersed in toluene are shown in Figure 1d. The PL spectrum was centered at 732 nm with a full width at half maximum (fwhm) of 154 nm. The large value of fwhm was consistent with the histogram of QD diameters and can be attributed to the polydisperse size of the De-SiQDs. The inset shows a toluene solution of De-SiQDs under the illumination of room light (left) and a 365-nm emitting handy lamp (right). The De-SiQDs were found to be highly dispersed in toluene, forming a transparent, uniform brown-color solution under room-illumination even at concentrations as high as 10 mg/mL. The measured value of absolute PLQY was 33%.

### 3.2. Si-iQLED Fabrication

**Figure 2:**
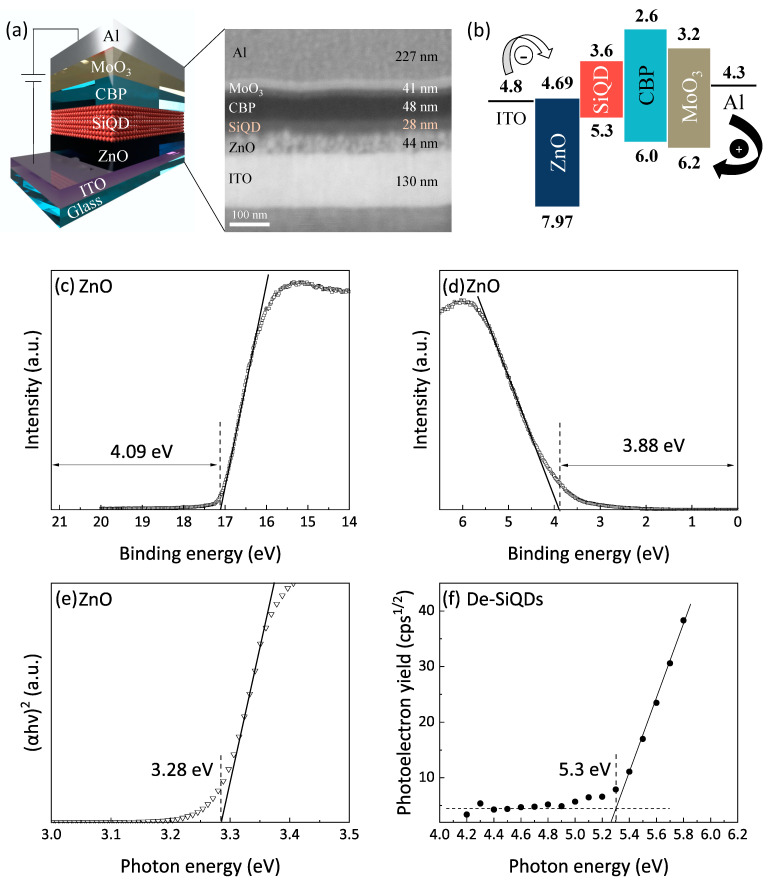
(**a**) Schematic illustration of Si-iQLEDs stack structure and a cross-sectional SEM image of the device structure with a hybrid organic/inorganic multilayer stack, (**b**) flat energy band diagram in the unbiased conditions. UPS spectra of (**c**) secondary-electron cutoff and (**d**) valence-band edge regions of ZnO thin film. (**e**) (αhν)^2^−hν plots converted from the absorption spectra of ZnO nanoparticles. (**f**) Photoelectron yield spectroscopic (PYS) spectrum of thin film of De-SiQDs.

Figure 2a represents the device architecture and its cross-sectional SEM image. In this work, the Si-QLED inverted device architecture (i.e., Si-iQLEDs) with an organic/inorganic hybrid multilayer was adopted. To predetermine the thickness of SiQD layer, we referred the previous work by Maier-flaig et al. who reported that the parasitic EL emissions from the neighboring compositional layer appeared when the QD-layer thickness is as thin as 11 nm, whereas a 31-nm QD layer was thick enough to disappear parasitic emission and improve luminance. Proposed energy level diagram under a zero applied bias voltage is shown in Figure 2b where the energy structures of ZnO and De-SiQD were characterized by UPS and PYS, respectively. The UPS spectra of the secondary electron cut-off and valence band edge regions of ZnO thin film are shown in Figure 2c,d. The work function calculated by the difference in energy between the incident light (21.2 eV) and the secondary-electron cutoff was 4.09 eV (see Figure 2c). The difference in energy between Fermi level and valence band maximum (VBM), which is extracted from the valence band edge region shown in Figure 2d, was estimated to be 3.88 eV. Thus, the calculated VBM level was approximately 7.97 eV from the vacuum level. The UV-vis spectrum of ZnO thin film shown in Figure 2e exhibited a plot of (αhν)^2^ versus photon energy (hν) and indicated a 3.28 eV of optical bandgap. Thus, the calculated conduction band minimum (CBM) was 4.69 eV as illustrated in Figure 2b. The PYS spectrum shown in Figure 2f indicated a 5.3 eV of VBM level for the De-SiQD film. On the assumption that the PL photon energy (1.7 eV) corresponds to the magnitude of optical bandgap, the CBM level was estimated to be 3.6 eV. The values of highest occupied molecular orbital (HOMO) and lowest unoccupied molecular orbital (LUMO) of the CBP layer and the VBM and CBM of MoO_3_ layer were taken from literature [6]. In Figure 2b, the low energy barrier between ITO and ZnO was suitable for efficient electron injection from the electrode at a low applied bias voltage. On the other hand, the deep HOMO energy level of ZnO worked as a hole blocking barrier, which confined holes within the De-SiQD layer. Furthermore, the high LUMO energy barrier of CBP layer worked to block a leakage of electrons to the adjacent anode, leading to the enhanced radiative electron-hole recombination in the De-SiQD layer for emission of light.

### 3.3. Device Performance of Si-iQLED

**Figure 3:**
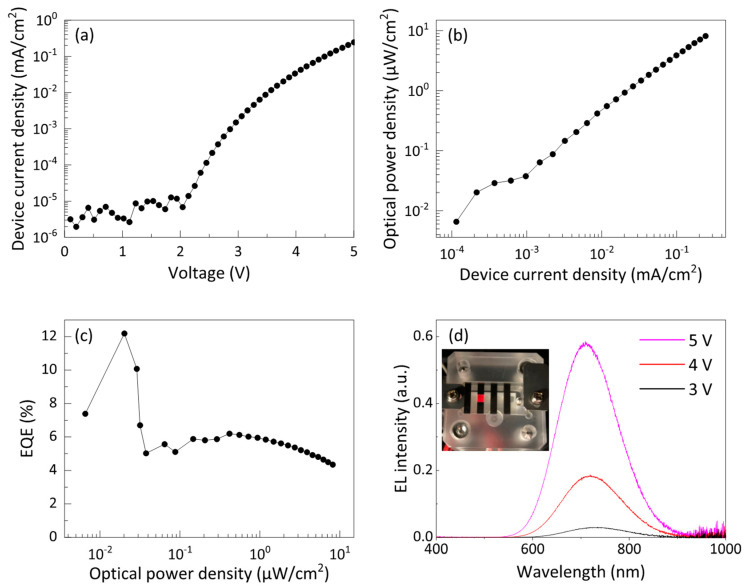
The device characteristics of Si-QLEDs of (**a**) *I-V* characteristics, (**b**) optical power density plotted by device current density, (**c**) EQE performance with optical power density, and (**d**) electroluminescence (EL) spectra operated at different voltages with an inset of device photograph operating at 5 V.

Instead of coating the surface of the QDs with short ligands during the hydrosilylation process, we found that applying a constant bias voltage of 5 V to the pristine Si-iQLEDs for a certain time (e.g., 9 h) shortened the interparticle distance between adjacent QDs in the emitting layer, as described below. This postproduction treatment was named “post-electric-annealing”. Figure S1a and Figure 3a show the current–voltage characteristics before and after electric annealing, respectively. The estimated turn-on voltage before electric annealing was 3.47 V, but after electric annealing, the turn-on voltage decreased to 2.45 V. Figure 3b shows optical power density characteristic versus device current density. The reason for using optical power density instead of luminance is that there is almost no visible sensitivity curve at EL peak wavelengths above 700 nm in the International Commission on Illumination (CIE) optical radiation efficiency function, which is used to convert radiant energy to luminance. The magnitude of the optical power density was found to increase significantly in the narrow range of 0.1–0.4 μA/cm^2^, with very small increases in the range of 0.4–1.0 μA/cm^2^ and gradual increases in device current density above 1 μA/cm^2^. The device exhibited an optical power density of up to 8.16 μW/cm^2^ at 5 V. On the other hand, the device before electric annealing showed a maximum optical power density of 6.63 μW/cm^2^ at 5 V (see Figure S1a). In Figure S1b and Figure 3c, the estimated EQE values before and after electric annealing are plotted as a function of optical power density. The maximum EQE before electric annealing was 1.18%. On the other hand, as expected from the optical power plot, the best EQE value after electric annealing was 12.2% at 0.02 μW/cm^2^, corresponding to the device current density range of 0.1–0.3 μA/cm^2^ in Figure 3b. Moreover, EQE exceeding 10% was reproducible (see Figure S2, Supplementary Material). Thereafter, as in the other case, the EQE decreased but remained higher than 4% even at 8.16 μW/cm^2^. Figure S3 compares the optical power density of Si-iQLEDs with other reported values [5,13,17,42]. Our optical power density (i.e., 8.16 μW/cm^2^) was lower than the reported values, but it was bright enough that the luminescent color could be seen with the naked eye under room lighting (see Figure 3d, inset). The low optical power density was perhaps caused by low device current density compared to the reported devices although the mechanism remains under investigation. Figure 3d shows EL spectra when the applied bias voltage was varied from 3 to 5 V. EL spectra with high signal-to-noise ratio were observed for all devices. The EL intensity increased with increasing voltage; the center of the EL spectrum at 3 V was at 730 nm, but it shifted to 710 nm at 5 V. Spectral blueshift of 20 nm, about 30 nm smaller than conventional Si-QLEDs [5,8,10,11,42], probably due to quantum confinement Stark effect and band filling effect at higher bias, applied voltage [43]. The absence of parasitic emission from the adjacent ZnO layer suggests that carriers did not leak from the QD layer to the adjacent ZnO and CBP layers even during high-voltage operation that promotes electron-hole recombination in the emitting layer for EL. Furthermore, the PL and EL spectra are overlapped exactly (see Figure S4, Supplementary Material), suggesting that the luminous characteristics is not influence by the distance of the SiQDs due to a large Stokes shift between the optical absorption and PL emission spectrum as evidenced in Figure 1d.

## 4. Discussion

**Figure 4:**
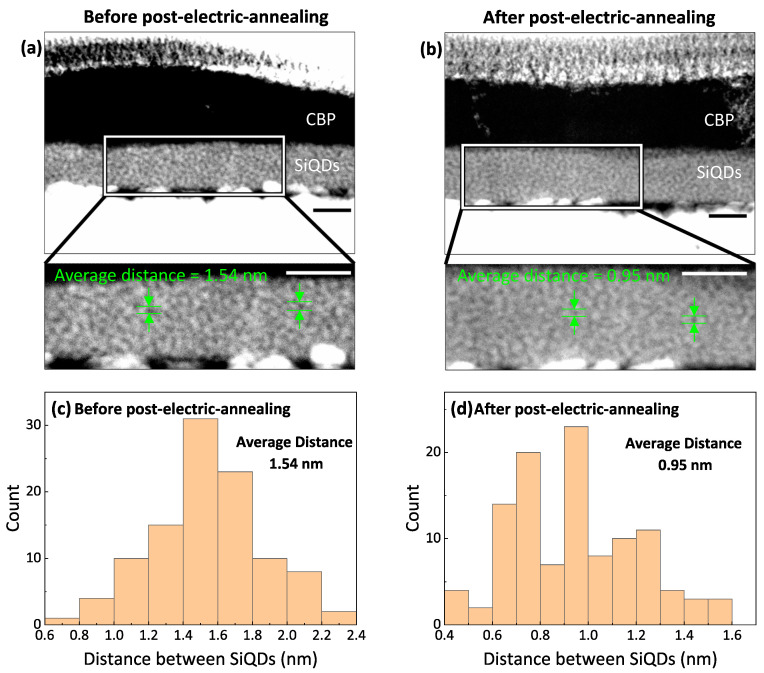
Cross-sectional HAADF-STEM images of Si-QLEDs and histogram of distance between SiQDs in the active layer (**a,c**) before and (**b**,**d**) after post-electric-annealing. For post-electric-annealing, the device was applied voltage for 22 h at 5 V. Each scale bar is 20 nm.

**Figure 5:**
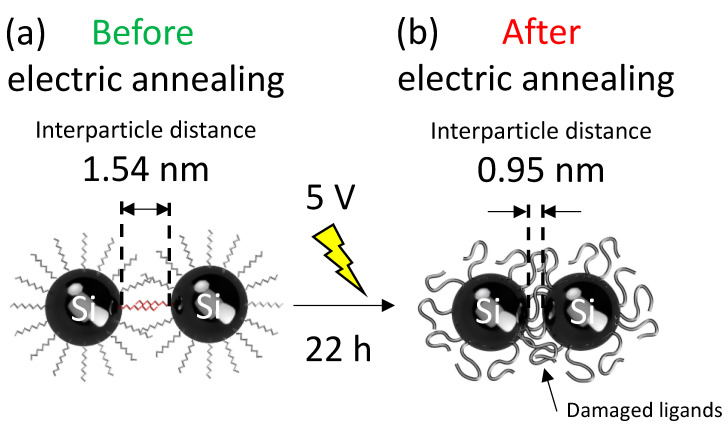
Schematic representation of electric-annealing mechanism in SiQDs layer at (**a**) before and (**b**) after electric-annealing state.

To clarify what is happening inside the device after electric annealing, another red-emitting Si-iQLED was fabricated and observed with electron microscopes. Figure 4a,b show cross-sectional HAADF-STEM images of the device before and after 22 h of voltage application. Focusing on the SiQDs layer, histograms of interparticle distances based on the cross-sectional views are shown in Figure 4a,b. The SiQDs stacks were clearly denser in both images, but the average distance between SiQDs before and after electrical annealing was estimated to be 1.54 nm and 0.95 nm, respectively. Based on these results, the possible mechanism of shortening the interparticle distance is shown in Figure 5. (1) Before voltage application, SiQDs assemble to form an active layer, but the capping ligands of neighboring QDs create steric hindrance and do not increase packing density as illustrated in Figure 5a. (2) When a voltage of 5 V is applied to the device, Joule heat is generated by the voltage application. As the voltage application time increases, the capping ligands begin to degrade due to Joule heat. As the ligands are carbonized due to thermal degradation, steric hindrance decreases and the interparticle distance between QDs is shortened, as illustrated in Figure 5b. Due to carbonized ligands and shortened interparticle distance, the device resistance decreases, leading to large current density. As a result, the radiative recombination rate within the SiQDs increases. (3) As the voltage application time increases, the decrease in the distance between SiQDs almost saturates, and so do the current density and optical power density of the device. (4) Finally, the further degraded ligands began to act as energy barriers within the SiQD layer (although further study is needed), reducing the current density and optical power density of the device. One of the concerns during electric annealing was a decrease in QD diameter, possibly due to oxidation or carbonization of Si surface. To investigate the influence on QD size under applied voltage application, EL spectra were measured at 5V in 1h-increments. As shown in Figure S5 (Supplementary Material), there were no changes in spectral shape and position of EL peaks. The results suggest that the surface of the OD is not oxidized or carbonized, or if it is, it is negligible. Moreover, the shortened interparticle distance did not influence on EL spectral shape and position, possibly due to no or less energy transfer between adjacent QDs in the emitting layer based on a large Stokes shift between absorption and PL spectra characteristic of SiQD.

**Figure 6:**
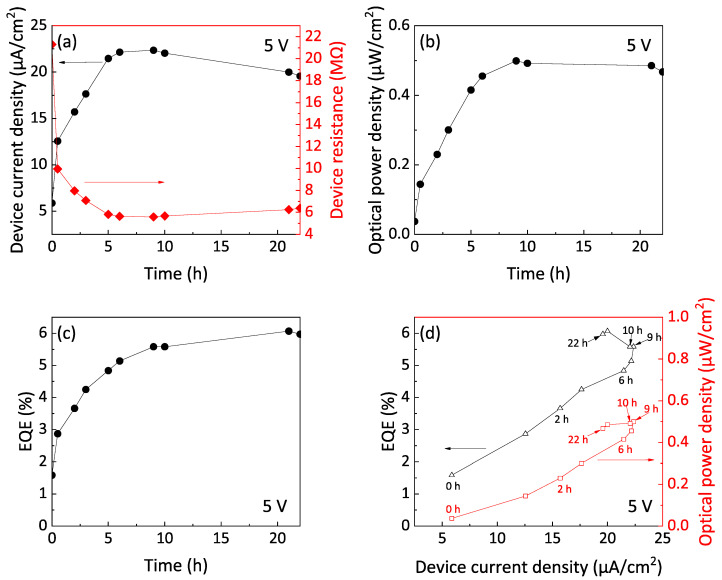
Device characteristics of (**a**) device current density and device resistance, (**b**) optical power, and (**c**) external quantum efficiency (EQE) plotted with time of voltage application. (**d**) The EQE and optical power plots with device current density during voltage application up to 22 h. Here, a constant voltage of 5 V was applied into the device.

Next, the device characteristics during electrical annealing were evaluated. Figure 6 shows a summary of changes in device performance with annealing time at 5 V. Changes in both device current density and device resistance with annealing time are shown in Figure 6a. During the first 6 h, device resistance decreased significantly, and current density increased; from 6 to 9 h, both current density and resistance were nearly saturated; after 10 h, resistance increased, and current density began to decrease. As shown in Figure 6b, the optical power density increased for the first 9 h, the optical power density almost saturated from 10 to 21 h, and the optical power density began to decrease after 22 h. During the electronic annealing, there was no change in spectral shape or peak position of the EL spectra (see Figure S5, Supplementary Material). Changes in EQE of Si-iQLED are shown in Figure 6c. The values of EQE increased for the first 21 h but began to decrease thereafter. Figure 6d shows the change over time of EQE and optical power density versus current density. From Figure 6d, it could be seen that the EQE increases with increasing current density up to 9 h. From 10 to 21 h, the current density decreased, but the EQE increased. On the other hand, the optical power density also increased with increasing current density up to 9 h. Interestingly, even as the current density decreased, the optical power density remained almost constant from 10 to 21 h, resulting in an increase in the EQE.

We also evaluated the luminous stability before and after cooling the device. Here, since electrical annealing also occurs with repeated application of voltage, the process was performed with repeated application of 5 V instead of applying a constant voltage. Figure S6 shows the changes in device current density and optical power density before and after cooling. The detail is described in Supplementary Material. After cooling the device, the first measurements of optical power density for all cycles (indicated by red arrows in Figure S6) dropped more rapidly than before cooling (indicated by black arrows in Figure S6). Thereafter, the optical power density increased with increasing applied voltage but did not reach the optical power density before cooling. These trends were also evident in the device current density. Therefore, it was determined that the decrease in optical power density was mainly due to the increase in the device resistance resulting from device degradation. From the above, it can be said that effective use of post-electric-annealing should be terminated at the point where device resistance is lowest and luminance is at its maximum. Otherwise, if the annealing time is further extended from that point, the external quantum efficiency will increase but luminance will decrease, and the device will begin to deteriorate as a light emitting device.

## 5. Conclusions

Diamond cubic Si is a material cornerstone for microelectronics industry. Photoluminescence of Si nanostructures was first reported by Canham [23]. By inspiring the pioneering work, the researchers have developed various synthetic approaches and the synthesized Si nanocrystals have been used as light-emitting and absorbing layers for optoelectronic applications [44]. For Si-QLEDs, it is desired to achieve the industry benchmark of 20% of EQE. Herein, we reported a new method, named post-electric-annealing, to improve EQE of red-emitting Si-iQLEDs. In our conditions, after 9 h of voltage application of 5 V, EQE was increased up to 12.2%. In this article, a constant voltage of 5 V was applied to the device for a fixed time. However, the method of applying the voltage is not limited to this way; the EQE could also be increased up to ~12% by repeatedly applying a voltage between 0 V and 5 V during a similar duration of time. Detailed transmission microscopic study revealed that the interparticle distance between adjacent De-SiQDs shortened in the emitting layer, facilitating charge carrier injection into the QDs layer and improving the photocurrent density and EQE. This phenomenon of interparticle distance shortening was assumed to be caused by ligand damage due to Joule heat generated during voltage application. To prevent degradation as a light emitting device, it was important to terminate the postproduction treatment at the position where the device resistance was the lowest and luminance was maximum. While the process is difficult to exploit in industry, the findings suggest that both shortening the interparticle distance between adjacent SiQDs in the optically active layer and improved packing density of QDs promises to improve the EQE by at least 12.2%.

## Data Availability

Not applicable.

## Supplementary Materials

The following supporting information can be downloaded at: https://www.mdpi.com/article/10.3390/nano12234314/s1, Figure S1: Device current density, optical power density and EQE performance before electrical annealing; Figure S2: Histograms of maximum EQEs of four Si-QLEDs; Figure S3: A summary of the optical power density of Si-QLEDs emitting in the same wavelength range as in this study; Figure S4: The comparison of PL and EL spectra operated at 3 V; Figure S5: Normalized EL spectra during annealing plotted in 1-hour increments at 5 V; Figure S6: Stability of the device current density and the optical power density before and after cooling the device; References [5,13,17,42] were cited in Supplementary Materials.
