# Supplementary material for: Postproduction Approach to Enhance the External Quantum Efficiency for Red Light-Emitting Diodes Based on Silicon Nanocrystals
Source: Nanomaterials (Basel). 2022 Dec 5;12(23):4314. doi: 10.3390/nano12234314 (PMC9735803; doi:10.3390/nano12234314)
Supplement: Supplementary file 1 [file nanomaterials-12-04314-s001.zip › nanomaterials-2055988-supplementary.pdf]

Supplementary Material

Postproduction Approach to Enhance the External Quantum Efficiency for Red Light-Emitting Diodes Based on Silicon Nanocrystals

Hiroyuki Yamada [1,2], Junpei Watanabe [1,3], Kazuhiro Nemoto [1,2], Hong-Tao Sun [1] and Naoto Shirahata [1,2,3,*]

[1] International Center for Materials Nanoarchitectonics (MANA), National Institute for Materials Science (NIMS), 1-1 Namiki, Tsukuba 305-0044, Japan
[2] Graduate School of Chemical Sciences and Engineering, Hokkaido University, Sapporo 060-0814, Japan
[3] Department of Physics, Chuo University, 1-13-27 Kasuga, Bunkyo, Tokyo 112-8551, Japan
* Correspondence: shirahata.naoto@nims.go.jp; Tel.: +81-29-859-2743

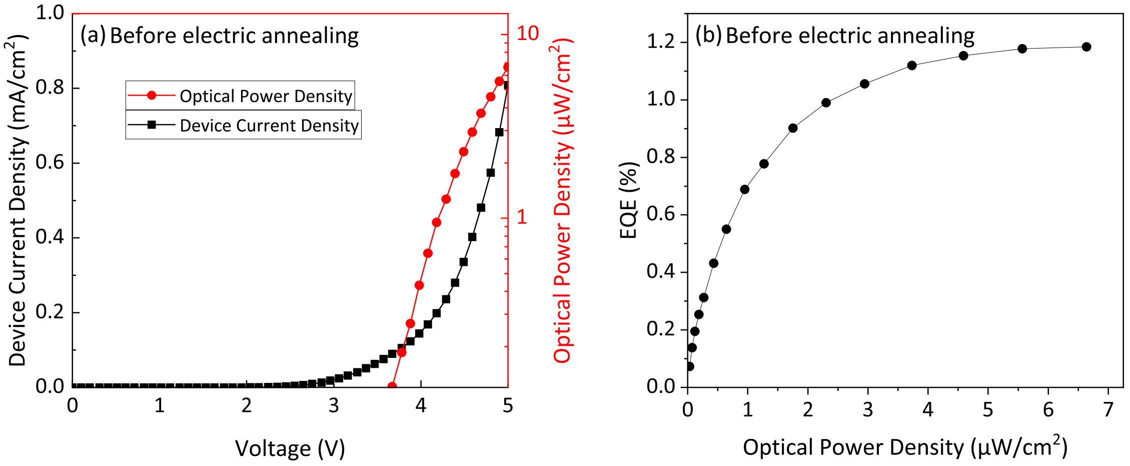

Figure S1. (a) Device current density and optical power density and (b) EQE performance before electric annealing.

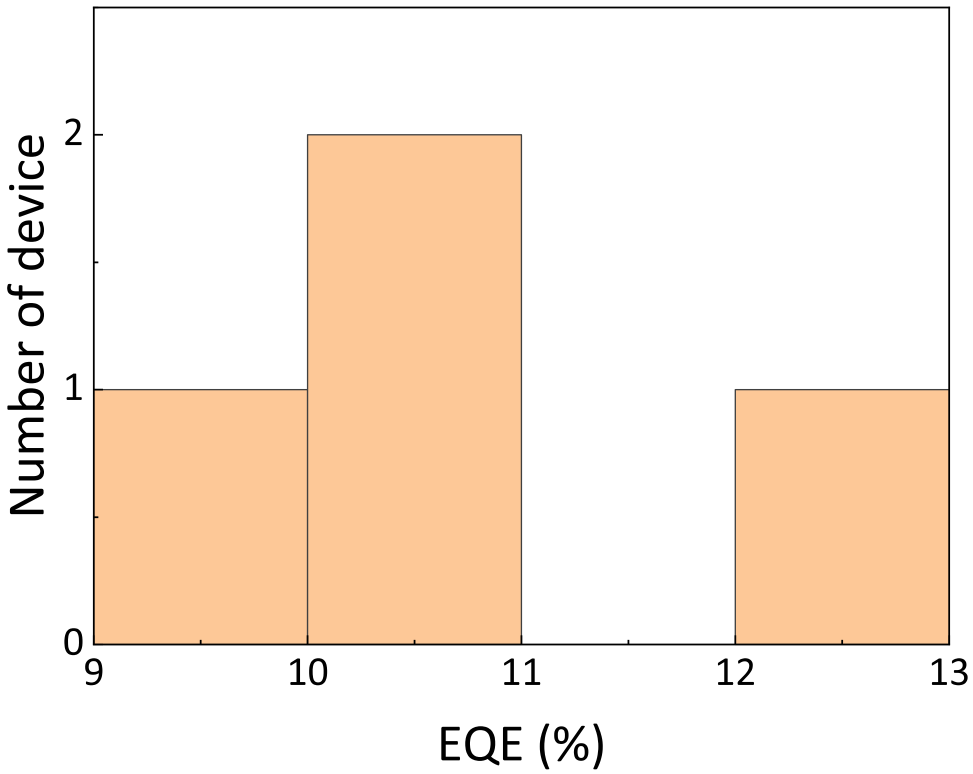

Figure S2. Histograms of maximum EQEs of four Si-QLEDs. The respective EQEs were 9.27%, 10.13%, 10.19%, and 12.2%.

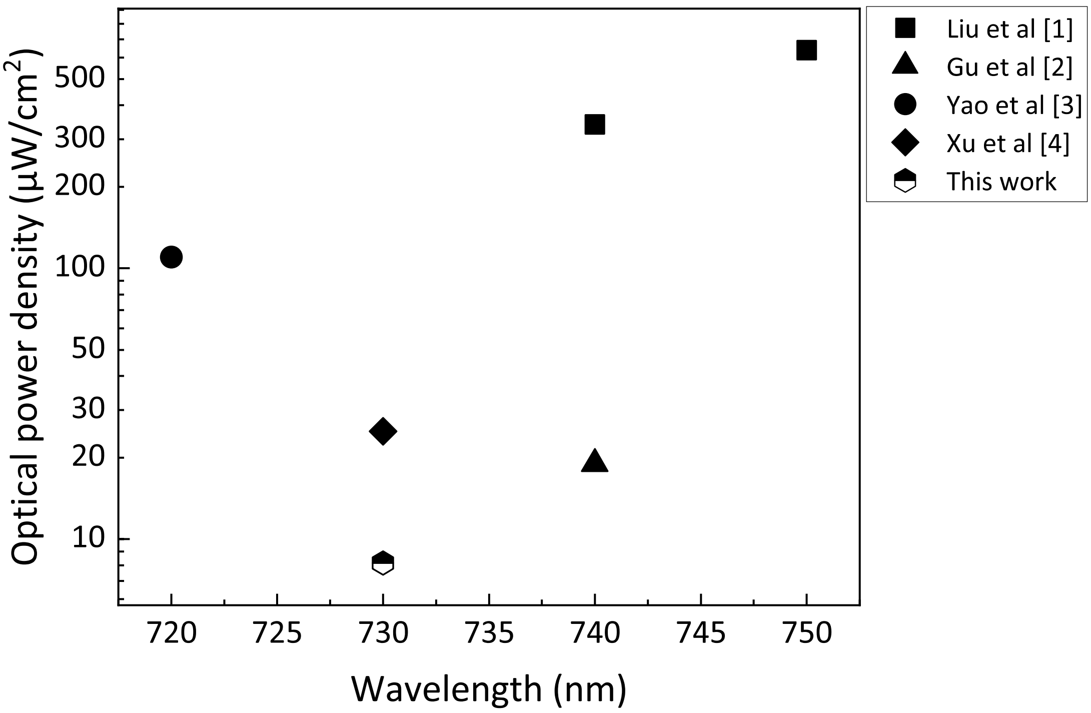

Figure S3. A summary of the optical power density of Si-QLEDs emitting in the same wavelength range as in this study [1–4].

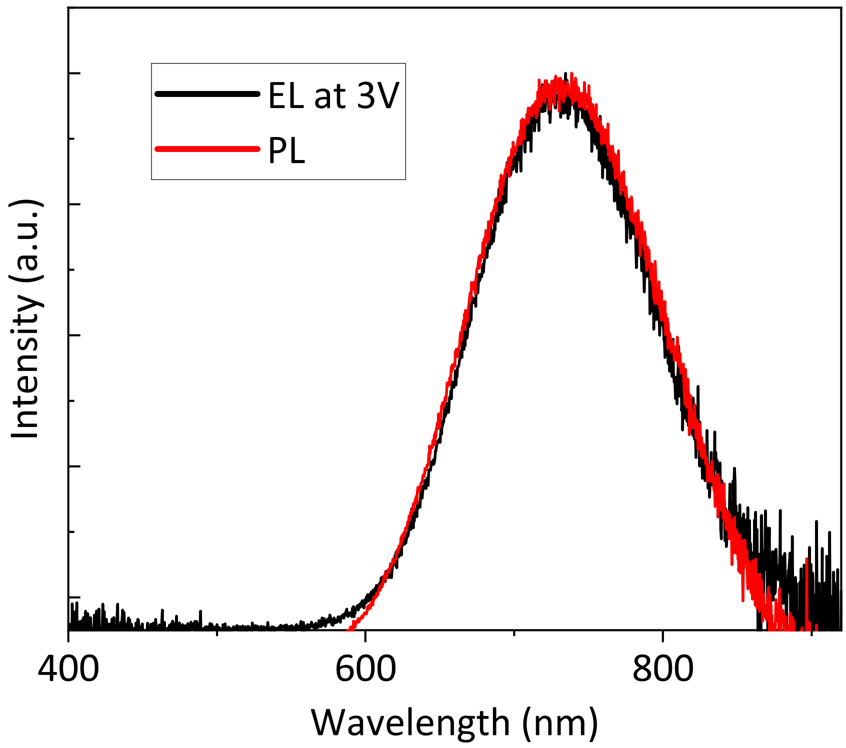

Figure S4. The comparison of PL and EL spectra operated at 3 V.

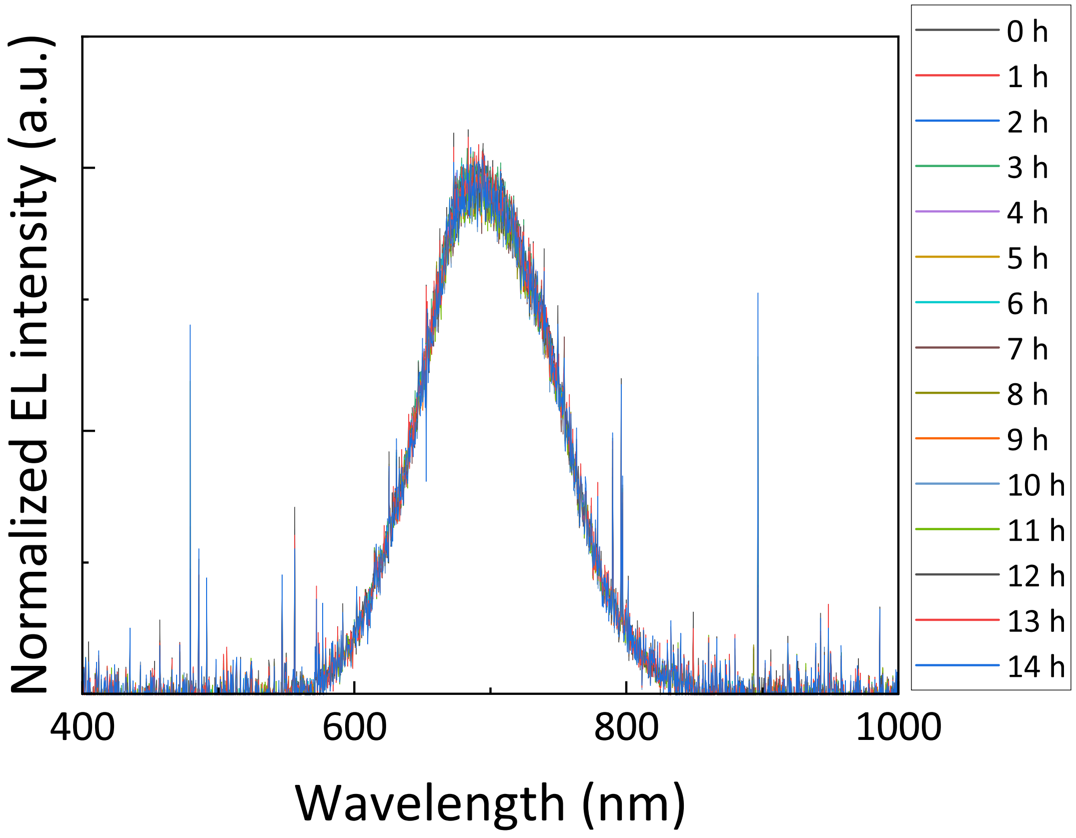

Figure S5. Normalized EL spectra during annealing plotted in 1-hour increments at 5 V.

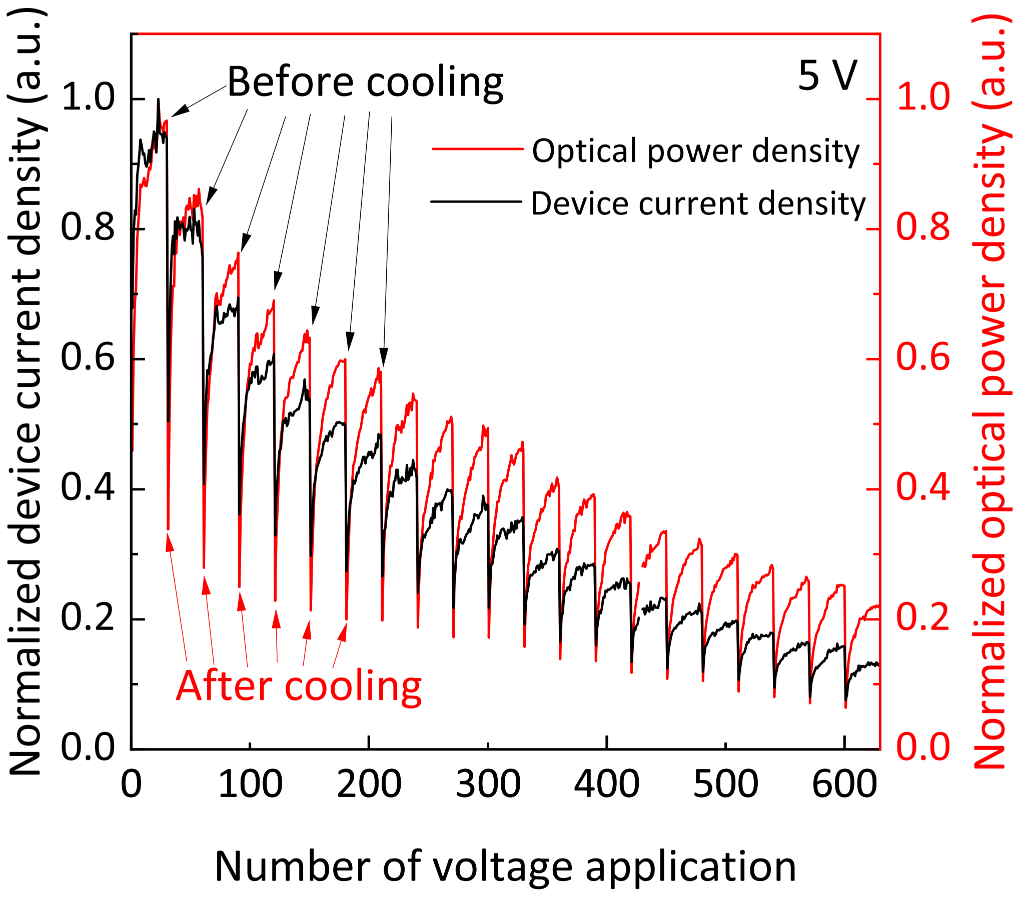

Figure S6. Stability of the device current density and the optical power density before and after cooling the device.

Here, the bias voltage was increased to 5 V in 51 steps, kept for 0.5 sec, and then returned to 0 V. This was repeated 30 times. Intervals between each repetition were 5 sec. After 30 times of measurement, turn off the power for 1 hour to cooling down the device. After 1 hour, the device is subjected to another 30 times of repetitive voltage applications again. This series of cycles was performed 21 times. From Figure S6, after the device cooling, every first measurement of optical power density of all cycles drops more sharply than that before cooling. After that, the optical power density tends to increase as

the number of applied voltages increases, but it does not reach the optical power density before cooling. These trends also appeared in the device current density. Therefore, it was concluded that the decrease in the optical power density was mainly due to the increase in the device resistance due to the degradation of the device.

Reference:

[1] Liu, X. *et al.* Light-Emitting Diodes Based on Colloidal Silicon Quantum Dots with Octyl and Phenylpropyl Ligands. *ACS Appl. Mater. Interfaces* **10**, 5959–5966 (2018)

[2] Gu, W. *et al.* Silicon-Quantum-Dot Light-Emitting Diodes With Interlayer-Enhanced Hole Transport. *IEEE Photonics J.* **9**, 4500610 (2017)

[3] Yao, L. *et al.* Efficient silicon quantum dots light emitting diodes with an inverted device structure. *J. Mater. Chem. C* **4**, 673–677 (2016)

[4] Xu, Y., Terada, S., Xin, Y., Ueda, H. & Saitow, K. Ligand Effects on Photoluminescence and Electroluminescence of Silicon Quantum Dots for Light-Emitting Diodes. *ACS Appl. Nano Mater.* **5**, 7787–7797 (2022)
